# Occurrence of Malnutrition among Seniors in Poland Depending on the Place of Residence: An Analysis of Socioeconomic and Health Risk Factors

**DOI:** 10.3390/nu16193394

**Published:** 2024-10-06

**Authors:** Iwona Klisowska, Anna Felińczak, Beata Jankowska-Polańska

**Affiliations:** 1 Department of Pediatrics and Coordinated Child Care, Department of Nursing, Faculty of Nursing and Midwifery, Wrocław Medical University, 51-618 Wrocław, Poland; iwona.klisowska@umw.edu.pl (I.K.); anna.felinczak@umw.edu.pl (A.F.); 2 Faculty of Medicine, Wrocław University of Science and Technology, 50-376 Wrocław, Poland

**Keywords:** malnutrition, seniors, place of residence, care institutions, quality of life, functional fitness, depression, frailty syndrome

## Abstract

The aging population in Poland poses significant socioeconomic and health challenges, particularly regarding malnutrition among seniors. This study examines the impact of place of residence on the nutritional status and related health outcomes of older adults. Data were collected from 338 community-dwelling seniors and those in long-term care facilities. The results indicate that long-term care residents exhibited significantly higher frailty and depression levels and poorer nutritional status, functional fitness, gait, and balance compared to those in communities. Self-reported quality of life did not differ significantly between groups. Regardless of residence, having a family correlated with better nutritional status, quality of life, and functional fitness and lower frailty and depression levels. Malnutrition was significantly associated with reduced functional fitness across all residences, and well-nourished individuals in care facilities had lower functional fitness than those who were at home. Community-dwelling residents had significantly lower frailty levels, with frailty negatively correlating with nutritional status. Normal nutritional status was linked to higher balance and gait scores, indicating a lower fall risk, with the risk further reduced for those living in community settings. Additionally, normal nutritional status correlated with lower depression levels and higher quality of life, with malnourished individuals experiencing better quality of life in community-dwelling settings. These findings underscore the critical role of residence and family support in elderly nutrition and health outcomes.

## 1. Introduction

Malnutrition among the elderly is considered one of the key geriatric issues due to its high prevalence, complex causes, and serious health consequences. World Health Organization (WHO) data indicate that the percentage of people over the age of 60 will increase from 12% in 2015 to 22% in 2050 [1]. Eurostat data from 2017 show that 19% of the EU population has surpassed 65 years of age. In Poland, the majority of people over 70 years old live in rural areas. In 2022, the total number of people over 60 years old was 9,797,710, of which 4,839,901 were over 70 years old [2].

The aging of the population has serious economic, social, and medical consequences. Older patients often suffer from multiple diseases simultaneously, which is associated with polypathology and polypharmacotherapy, as well as a lack of specificity in clinical symptoms [3]. Increasingly, attention is being drawn to nutritional disorders such as obesity and malnutrition, which are common iatrogenic complications of hospital therapies and chronic diseases [4].

Among older adults, the aging process leads to disturbances in the sensation of satiety, poorer compensation for the energy content of foods, and a weakening of the senses of smell and taste. As a result of reduced physical activity and a slower metabolic rate, there is a decrease in energy expenditure. All this leads to changes in the sensation of hunger and satiety, as well as a reduction in energy intake. However, the reduction in energy requirements is associated with a much higher risk of nutritional deficiencies, particularly in proteins, vitamins, and minerals [5].

Unfavorable socioeconomic factors contributing to malnutrition include poor financial situation, social isolation, loneliness, and periods of mourning [6]. Nutritional knowledge is also insufficient, often resulting in improper dietary behaviors in the elderly population [7]. Proper nutrition is one of the most crucial factors influencing the maintenance of health.

Despite growing interest in the issue of malnutrition, sources indicate that only 3 to 5% of patients undergo examinations aimed at diagnosing abnormal body weight, while the real problem affects about 30–60% of hospitalized patients and 85% of patients in long-term care institutions [8]. Malnutrition is often accompanied by other geriatric syndromes, functional limitations, and an increased risk of death; however, this problem is often not recognized, especially in hospitalized individuals and those in long-term care facilities. Malnutrition can cause a decrease in muscle mass, affecting not just skeletal muscles but also the heart muscle, resulting in decreased cardiac output and subsequent reduced kidney perfusion [9]. It negatively impacts the digestive system, leading to structural and functional disturbances in the intestines and pancreas.

Malnourished patients are more susceptible to infections and impaired wound healing [10]. Stratton et al. in a 2018 report estimated the cost of malnutrition at 19.6 billion pounds [11]. Additionally, an analysis of the risk factors for deaths related to Clostridioides difficile infection (CDI) based on comorbidities, demographic factors, and the care facility where the patient was hospitalized showed that the most significant risk factor for death was age over 84 years, which increases the likelihood by more than twenty-five times, followed by HIV infection, cancer, and malnutrition or abnormal weight loss [12].

The latest data from the annual Nutritional Care Tool report indicates that although many patients in healthcare facilities, mainly in hospitals and nursing homes, undergo screening, many of them (>40%) do not receive any form of nutritional support [13].

It is certain that malnutrition is a significant risk factor for both patient morbidity and increased healthcare costs through prolonged hospitalizations, morbidity, mortality, lower quality of life, higher risk of falls, and fractures. The lack of proper diagnosis of malnutrition in sick individuals can negatively impact the treatment process and cause a significant economic burden on society through prolonged hospitalizations or the need for institutionalization.

Despite great interest in the issue of malnutrition, there is still a lack of a universally accepted definition and a gold standard for conducting nutrition evaluation. According to current regulations in Poland, hospitals are required to assess the nutritional status of patients admitted to all hospital wards, except for emergency departments (ED) and with a few exceptions for 1–2 day stays, if the patient has not experienced weight loss in the past six months [14].

The situation of seniors in Poland is not optimistic. The currently changing family functioning model has resulted in senior individuals being left to live alone or, in the case of loss of independence, in institutional care [15]. Due to the lack of specific standards for diagnosing malnutrition among seniors living at home and those living in nursing homes and care facilities, it is crucial to understand the differences in the occurrence of malnutrition in this patient group depending on their place of residence.

The aim of the study was to identify the prevalence of malnutrition, including associated risk factors and health conditions among older adults, comparing community-dwelling with long-term care residents.

## 2. Materials and Methods

### 2.1. Research Tools

Standardized research tools were used in the study:

MNA (Mini Nutritional Assessment) Scale: This scale consists of two parts. Scoring no more than 7 points in the first part indicates malnutrition, 8 to 11 points indicates the risk of malnutrition, and 12 to 14 points indicates normal nutritional status. The second part of the scale involves patient assessment and includes questions about the number of medications taken daily, consumption of dairy products, fluid intake, and subjective assessment of nutritional status. Scoring 24 to 30 points indicates normal nutritional status, 17 to 23.5 points indicates a risk of malnutrition, and 17 points or less indicates malnutrition. The questionnaire is recommended by the American Society for Parenteral and Enteral Nutrition (ASPEN) and the European Society for Parenteral and Enteral Nutrition (ESPEN) [16].

ADL (Activities of Daily Living) Questionnaire: This tool assesses basic activities such as bathing/washing, dressing, using the toilet, transferring from/to the bed or chair, controlling urination and defecation, and eating without assistance. A score of 2 points indicates severe functional impairment and inability to function independently, 4 points indicates a moderate level of impairment, and 6 points indicates full normality [17].

IADL (Instrumental Activities of Daily Living) Questionnaire—Lawton Scale: This tool assesses complex daily activities such as managing finances, taking medications, housekeeping, meal preparation, using the telephone, and walking. It evaluates the degree of a patient’s dependence on a caregiver. A score of 27 points indicates independence, 26–10 points indicates the need for partial assistance, and 9 points or less indicates severe total dependence on others [18].

Edmonton Frailty Scale—This questionnaire assesses the presence and severity of frailty syndrome. The scale evaluates nine domains: clock drawing test for cognitive function, get-up-and-go test for balance and mobility, functional dependence, mood, medication use, social support, nutrition, continence, quality of life, and self-perceived health [19]. The maximum score is 17 points. The authors of the questionnaire have established the following scoring for frailty assessment:

0 to 4 points: no frailty
5 to 6 points: at risk of frailty
7 to 8 points: mild frailty
9 to 10 points: moderate frailty
11 or more points: severe frailty

Tinetti POMA (Performance Oriented Mobility Assessment) Test: This test assesses balance and gait and predicts the risk of falls. The maximum score of 28 points (16 points for balance and 12 points for gait) indicates normal function. A score below 19 points indicates a high risk of falls (risk increases fivefold), a score between 19 and 24 points indicates a tendency to fall, and a score above 24 points indicates a low but existing risk of falls [20].

WHOQoL-AGE Scale: This scale is dedicated to assessing the quality of life among older adults. Questions relate to satisfaction with personal relationships, friendships, energy levels, financial resources, the ability to meet needs, and satisfaction with achieving goals. Quality of life is scored from 0 to 100, with higher scores indicating better quality of life [21].

Geriatric Depression Scale—15-item version by Yesavage (GDS-15): This tool determines the severity of depressive symptoms in elderly patients. A score of 0 to 5 points indicates no depression, 6 to 10 points indicates moderate depression, and a score above 11 points indicates severe depression [22].

Simple anthropometric measurements were conducted in this study. The questionnaire interview covered topics such as the participant’s age, education, current chronic diseases, the number of medications taken, and the duration since the disease diagnosis, thus obtaining sociodemographic and clinical data. Multimorbidity was defined as the presence of three or more chronic diseases in a patient, and polypharmacy was defined as a situation in which a patient was taking at least five medications daily. The study was conducted by personnel trained in this area who conducted individual interviews with each patient. The abbreviations below are used throughout the study: MNA—Mini Nutritional Assessment; ADL—Activities of Daily Living; IADL—Instrumental Activities of Daily Living; FS—Edmonton Frailty Scale; Tinetti POMA—Tinetti Performance Oriented Mobility Assessment; WHOQOL-AGE—World Health Organization Quality of Life for Older Adults; GDS-15—Geriatric Depression Scale—15-item version by Yesavage.

### 2.2. Study Population

The study was conducted in institutional care facilities (*n* = 153) and the home environments of patients (*n* = 185). Before participating in the study, patients were informed about the purpose and procedures of the research. The study was conducted after obtaining written informed consent from each participant. Patients were assured of complete anonymity and were informed of their right to withdraw from the study at any stage.

A total of 463 patients who met the inclusion criteria were initially selected for the study; however, 338 patients ultimately completed all questionnaires and underwent anthropometric measurements. The inclusion criteria for the study group were age over 60 years and consent to participate in the study. The exclusion criteria were lack of consent to participate and diagnosed severe cognitive impairments.

This study was conducted individually with each patient between June 2019 and May 2021. The research involved anthropometric measurements and interviews using standardized research tools. Body composition analysis was performed using a Tanita scale, measurements were taken with a measuring tape, and then each patient participated in an interview during which questions from standardized questionnaires were read aloud, and their answers were entered into a database created for this study in the Statistica program. Participants also filled out a questionnaire designed for this study to gather sociodemographic factors and the patient’s medical history. As part of the project, no medicinal and/or diagnostic substances were administered to the patients. Data collection was done without the use of medical records. All activities were performed in person. The study locations included institutional care facilities: nursing care facilities (*n* = 39), social welfare homes (*n* = 18), preventive and therapeutic facilities (*n* = 96), and the patients’ home environments (*n* = 185) in the city of Wroclaw. Potential participants in institutional care were approached by research personnel in collaboration with the institutions’ staff. For community-dwelling individuals, recruitment was done after an initial expression of interest in participating, at which point a nurse visited them at home. During the home visit, the nurse provided detailed information about the study, and participants completed the questionnaire.

The study received approval from the Bioethics Committee of the Medical University of Silesia in Wrocław (Approval No. KB—124/2019).

### 2.3. Statistical Analysis

The data were collected in a database specifically made for this research in Statistica and then analyzed using SPSS 26.0 software.

Significance analyses were preceded by the calculation of descriptive statistics for indicators of nutrition, functional fitness level, frailty level, balance and gait assessment, depression, and quality of life. To determine the shape of the obtained distributions, statistics such as range (min.–max.), measures of central tendency (mean), dispersion (standard deviation), measures of skewness and kurtosis (skewness, kurtosis), and normality tests were calculated. To check whether the obtained distributions differed from the theoretical normal distribution, the Kolmogorov–Smirnov tests were calculated, as suggested for relatively large sample sizes [23]. The obtained statistical values showed that all analyzed indicators had statistically significant deviations from the normal distribution. The measures of asymmetry did not indicate significant skewness or kurtosis for most variables. However, a pronounced platykurtosis was observed in the balance and gait assessment results, suggesting a relatively large dispersion of values around the mean.

To determine the relationship between nutritional status, functional fitness, frailty level, balance and gait assessment, depression level, quality of life with age, and anthropometric parameters, the nonparametric Spearman’s rho correlation test was used. In assessing the relationship of the aforementioned variables with the duration of illness and the number of medications taken, Kendall’s tau-b test was applied. For intergroup comparisons, the Kruskal–Wallis H test was used. Additionally, post hoc comparisons were conducted using the Bonferroni–Dunn test, and a series of multifactorial variance analyses in a 3 × 2 scheme were performed to check whether the two factors, place of residence and nutritional level, interactively differentiated the functioning of seniors.

## 3. Results

Statistically significant differences were observed between those living in private households and those residing in care facilities in terms of education level. In private households, the highest percentages of individuals had primary and secondary education (19.1%; *n* = 49; 14.4%), while the lowest percentage had vocational education (7.4%). In contrast, in care facilities, the smallest group was composed of individuals with higher education (5.9%).

Analyzing marital status, the largest group among those living in their own households was married individuals (29.4%), whereas in care facilities, widows and widowers predominated (20.3%). Among the respondents, regardless of place of residence, cardiovascular diseases were most frequently reported (42.1% in private households vs. 38.2% in care facilities).

Individuals residing in long-term care facilities were more likely to suffer from neurological diseases (27.9% vs. 15.6%; *p* = 0.0001) and musculoskeletal disorders (36.2% vs. 31.8%; *p* = 0.0004) compared to those living in private households. Polypharmacy was more frequently noted among patients from private households than those in care facilities (30.6% vs. 27.1%; *p* = 0.01). The results are presented in Table 1.

To test the hypotheses, an analysis was conducted to assess nutritional status, functional fitness, frailty level, depression, and quality of life among the study participants based on their place of residence. Differences were observed across all compared parameters except for quality of life. A normal nutritional status was more frequently observed in individuals living in their own households compared to those residing in care facilities (36.6% vs. 28.1%). Conversely, the risk of malnutrition was more frequently observed among residents of care facilities (17.2% vs. 7.7%); *p* = 0.0001. Residents of long-term care facilities more often exhibited severe and moderate ADL (Activities of Daily Living) impairment (19.4% vs. 12.9%), while those living in private households more frequently maintained full functional independence (40.9% vs. 25.9%); *p* = 0.0001. The analysis of frailty syndrome occurrence showed that it did not affect 34.7% of patients living in their own households versus 16.2% of residents in care facilities. Frailty syndrome was more frequently observed among residents of care institutions (17.4% vs. 11.7%); *p* = 0.0001. Similarly, the risk of falls was more commonly observed among patients in care facilities compared to those living at home (35.9% vs. 22.4%; *p* = 0.0001), as was the occurrence of depression (20.3% vs. 13.8%; *p* = 0.0004). Patients, regardless of their place of residence, had the same average self-assessment level of quality of life. The results are presented in Table 2.

Based on the comparison of groups differentiated by place of residence, it was observed that patients residing in long-term care facilities had significantly higher scores for the occurrence of frailty syndrome (7 vs. 4; *p* < 0.001) and depression (5 vs. 3; *p* < 0.002) and significantly lower scores for nutritional status (24 vs. 27; *p* < 0.001), functional fitness (24 vs. 31; *p* < 0.001), and the balance and gait assessment (10 vs. 22; *p* < 0.001). The groups did not differ significantly in the assessment of quality of life. The results are presented in Table 3.

We observed that individuals who declared having a family, regardless of their place of residence, obtained significantly higher scores for nutritional status (27 vs. 25; *p* < 0.001), better functional fitness (32 vs. 25; *p* < 0.001), proper balance and gait assessment (21 vs. 13; *p* < 0.001), and higher quality of life (44 vs. 42; *p* = 0.011) compared to those without a family. Similarly, these individuals showed lower scores for the severity of frailty syndrome (4 vs. 6; *p* < 0.001) and levels of depression (3 vs. 5; *p* = 0.003). The results are presented in Table 4.

A series of multifactorial variance analyses allowed for examining whether nutritional status and place of residence interactively differentiated the considered characteristics of senior functioning. The analyses were conducted in a 3 × 2 scheme, where the between-subject factors were nutritional status (malnutrition vs. risk of malnutrition vs. normal nutritional status) and place of residence. The first variance analysis model concerned functional fitness (Table S1).

The results showed that the main effect of the “place of residence” factor was not statistically significant: *F* = 2.61; *p* = 0.107; *η*^2^ = 0.006. This means that individuals living in community-dwelling settings and care facilities did not differ in terms of the severity of functional fitness. Statistically significant differences, however, emerged among individuals with different nutritional statuses: *F* = 49.87; *p* < 0.001; *η*^2^ = 0.240. Simple effects tests, considering the Bonferroni correction and comparing individual pairs of means, showed that malnourished individuals had significantly lower functional fitness compared to those at risk of malnutrition and those with normal nutritional status. Individuals at risk also exhibited significantly lower fitness than those with normal nutritional status. There was also a statistically significant interaction effect of place of residence vs. nutritional status: *F* = 3.74; *p* = 0.025; *η*^2^ = 0.018. Post-hoc tests in this case revealed that the differences described above regarding nutritional status also manifested among respondents living either in care facilities or communities. Interestingly, it was also found that well-nourished individuals residing in care facilities had lower functional fitness compared to those living in the community.

Analyses conducted for the frailty level indicator (Table S2) revealed a main effect of nutritional status similar to the one described above: *F* = 67.30; *p* < 0.001; *η*^2^ = 0.301. This effect indicated that the better the nutritional status, the lower the frailty level. The main effect of place of residence was also significant—individuals living in the community had significantly lower frailty: *F* = 5.70; *p* = 0.018; *η*^2^ = 0.013. The interaction effect of both factors was significant at the trend level: *F* = 2.67; *p* = 0.071; *η*^2^ = 0.012. The described intergroup differences regarding nutritional status also manifested among respondents living either in care facilities or communities. Well-nourished individuals residing in care facilities had a higher frailty level compared to those living in community-dwelling settings (Table S2).

Additionally, it was observed that within the group of well-nourished individuals, residents of long-term care facilities exhibited higher levels of frailty compared to those living in community-dwelling settings.

Regarding the overall balance and gait assessment indicator (Table S3), statistically significant differences were observed based on nutritional status (*F* = 39.66; *p* < 0.001; *η*^2^ = 0.193), where individuals with normal nutritional status achieved significantly higher scores than the other two groups. Moreover, it was observed that individuals living in community settings had higher scores than residents of care facilities: *F* = 20.45; *p* < 0.001; *η*^2^ = 0.050.

There was also a statistically significant interaction between the analyzed factors: *F* = 4.45; *p* = 0.012; *η*^2^ = 0.022. Post-hoc tests revealed that in both the group of residents of long-term care facilities and the group of individuals living in community settings, those with a normal nutritional status achieved significantly higher balance and gait scores, indicating a lower risk of falling compared to individuals with malnutrition or those at risk of malnutrition. Additionally, it was found that seniors at risk of malnutrition and those with a normal nutritional status exhibited a significantly lower risk of falling when living in community settings than when residing in care facilities.

In the next two-factor model of variance analysis, whether the place of residence and nutritional status differentiated the level of depression was examined (Table S3).

The obtained comparison results showed statistically significant differences among individuals with different nutritional statuses. The better the nutritional status, the lower the level of depression among the study participants: *F* = 19.88; *p* < 0.001; *η*^2^ = 0.114. However, no differences were observed based on place of residence: *F* = 0.04; *p* = 0.849; *η*^2^ = 0.000. The interaction effect was also not significant: *F* = 1.31; *p* = 0.271; *η*^2^ = 0.008. This means that individuals residing in long-term care facilities and communities, regardless of their nutritional status, did not differ significantly in terms of depression severity. The final analysis regarding differences in the level of quality of life is presented in Table S4.

In the case of quality of life, no significant differences were noted based on the place of residence (*F* = 1.90; *p* = 0.169; *η*^2^ = 0.005). However, it was again observed that individuals with normal nutritional status achieved higher quality of life scores compared to those at risk of malnutrition and those who were malnourished: *F* = 31.38; *p* < 0.001; *η*^2^ = 0.181. There was also a significant interaction effect at the trend level for both factors (*F* = 2.38; *p* = 0.095; *η*^2^ = 0.014) (Table S5). Bonferroni-corrected post-hoc tests revealed that malnourished individuals had a higher quality of life when living in the community. Furthermore, for participants residing in both long-term care facilities and in the community, those with normal nutritional status exhibited a higher quality of life compared to malnourished individuals and those at risk of malnutrition. The combined analysis of nutritional status, functional fitness, frailty, balance and gait, depression, and quality of life, based on the results of the multiple regression models, is presented in Table 5, providing a clear comparison of the key health indicators and their relationship with nutritional status across both community-dwelling seniors and long-term care residents.

## 4. Discussion

Our results have revealed significant differences in nutritional status, functional fitness, frailty level, balance and gait assessment, as well as depression and quality of life depending on the place of residence of seniors. Furthermore, it was confirmed that patients residing in long-term care facilities are more susceptible to malnutrition, have lower functional fitness, and have higher levels of frailty and depression compared to seniors living in a home environment. These findings are consistent with a recent meta-analysis that also indicated a higher prevalence of frailty in nursing homes and significant associations between frailty and various sociodemographic factors (living alone, poor self-rated health), physiological factors (poor sleep quality, low daily activity), behavioral factors (lack of physical activity), and disease-related factors (chronic diseases, depression) [24].

The nutritional status of seniors also proved to be a crucial factor affecting many key aspects of health. According to our results, inadequate nutrition led to a decrease in functional fitness, increasing the risk of falls and fractures, which aligns with the literature indicating a strong correlation between malnutrition and an increased risk of falls and injuries in the geriatric population [25]. Moreover, well-nourished individuals exhibited lower levels of depression and higher quality of life, highlighting the importance of appropriate nutritional support in improving the mental health of seniors.

The place of residence significantly impacted the nutritional status and related health parameters of seniors. Those living in the community showed better outcomes for functional fitness, balance and gait, frailty, and depression compared to residents of long-term care facilities. These results suggest that living in a home environment may promote better health and nutrition among seniors, potentially due to greater autonomy, better social support, and more individualized nutritional care.

Having a family also proved to be an important factor in supporting the health of seniors. This study showed that individuals with family had better nutritional status, improved functional fitness, lower levels of frailty, and reduced depression. These findings underscore the role of family support in maintaining the health of seniors, consistent with literature indicating the positive impact of social support on the health and well-being of older adults.

Analyzing data from the literature reveals that it is inconclusive, indicating that the problem of malnutrition affects approximately 5 to 30% of seniors living in a home environment [8]. Pol Senior studies point to an even higher percentage of poorly nourished individuals [26]. This potentially results from multifactorial issues with the diagnostic criteria used, and thus, further analyses of the nutritional status of older people living in their own households and those in institutional care are still needed, along with the preparation and implementation of programs aimed at improving nutritional status. It is important to consider that long-term care residents generally represent a sicker, frailer population with higher levels of comorbidities. Given these factors, one would expect malnutrition to be more prevalent in this setting.

The use of validated, quick, and easy-to-use research tools for assessing and detecting malnutrition in seniors can primarily expedite diagnosis and prevent its consequences. Lorenzo Donini’s analysis of the compliance of various tools showed that in screening tools (NRS, MUST, MNA, MNA-SF), a significant relationship between malnutrition and mortality can be assessed [27]. Additionally, the MNA scale presents the best predictive value for survival among well-nourished seniors [27]. Furthermore, factors determining functional and psychological fitness, which are not included in the MUST and NRS-2002 tools, are likely more important reasons for the risk of malnutrition than the disease itself [27].

This study has several limitations that should be considered when interpreting the results. First, the data were collected through self-reported questionnaires, which may introduce response bias, particularly among long-term care residents who might have underreported or exaggerated their health conditions in the hopes of receiving better care. Future studies should incorporate objective measures, such as clinical assessments or dietary logs, to validate self-reported data. Moreover, the findings of this study are based on a sample of older adults in one area of Poland and may not be generalizable to all populations. Statistical associations and correlations do not imply causality. Finally, while the study focused on nutritional status, other factors, such as cognitive function, social support, and physical activity, which may also significantly impact quality of life and frailty, were not thoroughly examined.

The study results indicate the need to intensify efforts to improve the nutritional status of seniors, especially those residing in long-term care facilities. This requires standardizing nutritional assessment procedures, raising nutritional awareness among seniors and their caregivers, and tailoring nutrition plans to individual patient needs. Additionally, it is important to increase the availability of supportive services for seniors living at home, which can contribute to improving their health and quality of life. Routine nutritional screening using validated tools like the Mini Nutritional Assessment should be implemented to identify at-risk individuals early. Personalized nutritional intervention, along with improved staff training on recognizing malnutrition, are crucial steps. Enhancing the quality and variety of meals, along with social interaction during mealtimes, can further improve nutritional outcomes. Finally, policymakers should establish guidelines requiring long-term care facilities to adhere to standardized nutritional assessments and care practices to ensure better health outcomes for residents.

## 5. Conclusions

In summary, this study highlighted significant relationships between nutritional status and various aspects of seniors’ health, emphasizing the importance of nutritional and social support. Further studies are needed, along with the introduction of measures to enhance the nutritional well-being and overall health of the elderly, which is vital for improving their quality of life.

## Figures and Tables

**Table 1:** Frequency distributions of sociodemographic characteristics in correlation with the place of residence. Statistically significant results (*p* < 0.05) have been indicated with a bolded font.

		Community-Dwelling		Care Facility		
		*n*	%	*n*	%	*p*
Sex	Men	69	20.3%	58	17.1%	0.85716
	Women	116	33.8%	95	27.9%	
Education	Primary	65	19.1%	41	12.1%	**0.00144**
	Vocational	25	7.4%	43	12.6%	
	Secondary	49	14.4%	50	14.7%	
	Higher	45	13.2%	20	5.9%	
Having a family	No	50	14.7%	135	39.7%	**0.00001**
	Yes	134	39.4%	19	5.6%	
Marital status	Single	13	3.8%	18	5.3%	**0.01184**
	Married	100	29.4%	58	17.1%	
	Widow/er	59	20.9%	78	22.9%	
For how many years has the disease/diseases been diagnosed	Up to 3	30	8.8%	18	5.3%	**0.0098**
	From 4 to 6	28	8.2%	28	8.2%	
	From 7 to 10	44	12.9%	53	15.6%	
	From 11 to 20	47	13.8%	28	8.2%	
	Above 20	17	5.0%	25	7.4%	
How many medications are taken daily	None	16	4.7%	7	2.1%	
	From 1 to 2	22	6.5%	13	3.8%	**0** **.01436**
	From 3 to 5	42	12.4%	42	12.4%	
	Above 5	104	30.6%	92	27.1%	
Chronic cardiovascular diseases	No	41	12.1%	23	6.8%	0.15841
	Yes	143	42.1%	130	38.2%	
Chronic respiratory diseases	No	120	35.3%	111	32.6%	0.11506
	Yes	64	18.8%	41	12.1%	
Chronic neurological diseases	No	131	38.5%	59	17.4%	**0.0001**
	Yes	53	15.6%	95	27.9%	
Chronic musculoskeletal diseases	No	76	22.4%	31	9.1%	**0.0004**
	Yes	108	31.8%	123	36.2%	

**Table 2:** Frequency distributions of characteristics considering nutrition, functional fitness level, frailty level, balance and gait assessment, depression, and quality of life according to place of residence. Statistically significant results (*p* < 0.05) have been indicated with a bolded font.

		Community-Dwelling		Care Facility		
		*n*	%	*n*	%	*p*
Nutritional status according to the MNA scale	Malnutrition < 17 points	20	5.7%	13	4.7%	**0.00001**
	Risk of malnutrition 17–23.5 points	27	7.7%	47	17.2%	
	Normal nutritional status 24–30 points	128	36.6%	77	28.1%	
Functional fitness level according to the ADL scale	Severe functional impairment—2 points	18	5.3%	21	6.2%	**0.00001**
	Moderate functional impairment—4 points	26	7.6%	45	13.2%	
	Fully preserved functions—6 points	139	40.9%	88	25.9%	
Frailty level (FS) according to the Edmonton Frailty Scale	No risk 0–4 points	118	34.7%	55	16.2%	**0.00001**
	Risk state FS 5–6 points	21	6.2%	32	9.4%	
	Mild FS 7–8 points	19	5.6%	30	8.8%	
	Moderate FS 9–10 points	11	3.2%	25	7.4%	
	Severe FS >11 points	10	2.9%	4	1.2%	
Balance and gait assessment according to the Tinetti test	High risk of falling <19 points	76	22.4%	122	35.9%	**0.00001**
	Moderate risk of falling 19–24 points	67	19.7%	26	7.6%	
	Low risk of falling >24 points	38	11.2%	5	1.5%	
Depression level according to the Yesavage scale	No depression 0–5 points	136	40.0%	86	25.3%	**0.00007**
	Moderate depression 6–10 points	33	9.7%	66	19.4%	
	Severe depression >11 points	14	4.1%	3	0.9%	
Quality of life level according to subjective assessment (WHOQOL-AGE)	Very good	6	3.5%	2	1.2%	0.09705
	Good	89	51.73%	756	46.8%	
	Neither good nor bad	57	3.1%	122	38.1%	
	Bad	16	9.3%	22	13.7%	
	Very bad	4	2.3%	0	0%	

**Table 3:** Nutritional status, functional fitness level, frailty level, balance and gait assessment, depression, and quality of life in correlation with place of residence. Mdn refers to the median, which represents the middle value of the data set. Mrang denotes the mean rank, a measure used to indicate the average ranking of observations, particularly in non-parametric tests. U is the U statistic from the Mann-Whitney U test, used to compare differences between two independent groups. *p* represents the *p*-value, which indicates the statistical significance of the results (with values less than 0.05 considered significant). Rg refers to the effect size, measured as rank-biserial correlation, which represents the strength of the relationship between the variables. Statistically significant results (*p* < 0.05) have been indicated with a bolded font.

	Community-Dwelling (*n* = 175)		Care Facility (*n* = 137)		*U*	*p*	*Rg*
	*Mdn*	*Mrang*	*Mdn*	*Mrang*			
Nutritional status	27.00	174.79	24.50	133.13	8786.00	**<0.001**	0.27
Functional fitness level	31.00	195.30	24.00	133.81	8729.00	**<0.001**	0.37
Level of frailty	4.00	136.12	7.00	195.96	8255.00	**<0.001**	0.37
Balance and gait assessment	22.00	207.94	10.00	119.66	6527.50	**<0.001**	0.53
Level of depression	3.00	154.49	5.00	187.22	11,436.00	**0.002**	0.19
Quality of life level	43.00	160.95	42.00	142.86	10,133.50	0.073	0.12

**Table 4:** Nutritional status, functional fitness level, frailty level, balance and gait assessment, depression, and quality of life in correlation with living with family. Mdn refers to the median, which represents the middle value of the data set. Mrang denotes the mean rank, a measure used to indicate the average ranking of observations, particularly in non-parametric tests. U is the U statistic from the Mann-Whitney U test, used to compare differences between two independent groups. *p* represents the *p*-value, which indicates the statistical significance of the results (with values less than 0.05 considered significant). Rg refers to the effect size, measured as rank-biserial correlation, which represents the strength of the relationship between the variables. Statistically significant results (*p* < 0.05) have been indicated with a bolded font.

	Living with Family						
	No (*n* = 168)		Yes (*n* = 144)				
	*Mdn*	*Mrang*	*Mdn*	*Mrang*	*U*	*p*	*Rg*
Nutritional status	25	138.46	27	177.54	9066.00	**<0.001**	0.25
Functional fitness level	25	143.65	32	194.81	9529.00	**<0.001**	0.31
Level of frailty	6	185.21	4	135.49	9026.50	**<0.001**	0.31
Balance and gait assessment	13	139.95	21	199.11	8775.00	**<0.001**	0.36
Level of depression	5	182.68	3	151.33	11,374.50	**0.003**	0.19
Quality of life level	42	139.82	44	165.57	9375.00	**0.011**	0.17

**Table 5:** Summary of multiple regression outcomes for nutritional status and health indicators across community-dwelling and long-term care residents. Nutritional status (A = malnutrition, B = risk of malnutrition, C = normal nutritional status) and place of residence (I = community-dwelling, II = long-term care) on health indicators such as functional fitness, frailty, balance and gait, depression, and quality of life. M (Mean) and SD (Standard Deviation) represent the average scores and variability for each group based on respective assessment scales. The F values indicate significant differences between groups, and *p* values reflect comparisons between community-dwelling and long-term care residents. η^2^ represents the effect size, indicating the proportion of variance explained by the factors. Post-hoc comparisons highlight significant differences between the groups (A < B < C and I < II).

Indicator	Malnutrition (Mean ± SD)	Risk of Malnutrition (Mean ± SD)	Normal Nutritional Status (Mean ± SD)	Community-Dwelling (Mean ± SD)	Long-Term Care (Mean ± SD)	Statistical Significance (*p*-value)	Post-Hoc Comparisons
Functional Fitness	17.97 ± 6.12	21.82 ± 6.93	28.38 ± 5.90	27.43 ± 7.22	23.54 ± 6.72	*p* < 0.001	A < B < C
Frailty Level	9.71 ± 3.15	7.53 ± 2.79	4.06 ± 2.85	4.50 ± 3.65	6.63 ± 2.95	*p* < 0.001	A < B < C
Balance and Gait (Tinetti)	9.30 ± 6.80	9.53 ± 7.36	18.51 ± 8.14	18.87 ± 8.16	11.01 ± 7.84	*p* < 0.001	A < B < C; I > II
Depression (Yesavage Scale)	7.06 ± 3.70	5.45 ± 3.50	3.58 ± 2.86	4.09 ± 3.54	4.75 ± 3.01	*p* < 0.001	A < B < C
Quality of Life (WHOQOL-AGE)	36.23 ± 6.91	37.96 ± 6.99	44.62 ± 6.98	42.90 ± 7.96	41.39 ± 7.42	*p* < 0.05	A < B < C

## Data Availability

The original contributions presented in the study are included in the article/supplementary material. Further inquiries can be directed to the corresponding author.

## Supplementary Materials

The following supporting information can be downloaded at: https://www.mdpi.com/article/10.3390/nu16193394/s1, Table S1: Nutritional status and place of residence in correlation with functional fitness level—multifactorial variance analysis; Table S2. Nutritional status and place of residence in correlation with the level of frailty—multifactorial variance analysis; Table S3. Nutritional status and place of residence in correlation with overall balance and gait—multifactorial variance analysis; Table S4. Nutritional status and place of residence in correlation with depression—multifactorial variance analysis; Table S5. Nutritional status and place of residence in correlation with quality of life—multifactorial variance analysis.

## References

[1] Ageing and Health. https://www.who.int/news-room/fact-sheets/detail/ageing-and-health.

[2] Statistical Analyses (2023). The Situation of Older People in Poland in 2022.

[3] Aggarwal P., Woolford S.J., Patel H.P. (2020). Multi-Morbidity and Polypharmacy in Older People: Challenges and Opportunities for Clinical Practice. Geriatrics.

[4] Phelps N.H., Singleton R.K., Zhou B., Heap R.A., Mishra A., Bennett J.E., Paciorek C.J., Lhoste V.P., Carrillo-Larco R.M., Stevens G.A. (2024). Worldwide Trends in Underweight and Obesity from 1990 to 2022: A Pooled Analysis of 3663 Population-Representative Studies with 222 Million Children, Adolescents, and Adults. Lancet.

[5] Kaur D., Rasane P., Singh J., Kaur S., Kumar V., Mahato D.K., Dey A., Dhawan K., Kumar S. (2019). Nutritional Inter-ventions for Elderly and Considerations for the Development of Geriatric Foods. Curr. Aging Sci..

[6] Starr K.N.P., McDonald S.R., Bales C.W. (2015). Nutritional Vulnerability in Older Adults: A Continuum of Concerns. Curr. Nutr. Rep..

[7] Ghalib H., Mahmood N. (2024). Understanding Healthy Eating Habits of Elderly People in a Geriatric Center in Kirkuk City: A Cross-Sectional Study. Cureus.

[8] Pierzak M., Szczukiewicz-Markowska G., Głuszek S. (2020). The Problem of Hospital Malnutrition and Its Consequences. Med. Stud./Stud. Med..

[9] Deferrari G., Cipriani A., La Porta E. (2021). Renal Dysfunction in Cardiovascular Diseases and Its Consequences. J. Nephrol..

[10] Seth I., Lim B., Cevik J., Gracias D., Chua M., Kenney P.S., Rozen W.M., Cuomo R. (2024). Impact of Nutrition on Skin Wound Healing and Aesthetic Outcomes: A Comprehensive Narrative Review. JPRAS Open.

[11] Stratton R., Smith T., Gabe S. (2018). Managing Malnutrition to Improve Lives and Save Money President Elect of BAPEN 3 President of BAPEN. https://www.bapen.org.uk/pdfs/reports/mag/managing-malnutrition.pdf.

[12] Dziennik Urzędowy Ministra Zdrowia. https://dziennikmz.mz.gov.pl/legalact/2021/69/.

[13] Saunders J., Smith T. (2010). Malnutrition: Causes and Consequences. Clin. Med..

[14] Rozporządzenie Ministra Zdrowia z Dnia 22 Listopada 2013 r. w Sprawie Świadczeń Gwarantowanych z Zakresu Leczenia Szpitalnego. https://isap.sejm.gov.pl/isap.nsf/DocDetails.xsp?id=WDU20130001520.

[15] Statistics Poland The Situation of Older People in Poland in 2021. https://stat.gov.pl/en/topics/older-people/older-people/the-situation-of-older-people-in-poland-in-2021,1,4.html.

[16] Vellas B., Guigoz Y., Garry P.J., Nourhashemi F., Bennahum D., Lauque S., Albarede J.L. (1999). The Mini Nutritional As-sessment (MNA) and Its Use in Grading the Nutritional State of Elderly Patients. Nutrition.

[17] Hettiarachchi J., Reijnierse E.M., Soh C.H., Agius B., Fetterplace K., Lim W.K., Maier A.B. (2021). Malnutrition Is Associated with Poor Trajectories of Activities of Daily Living in Geriatric Rehabilitation Inpatients: RESORT. Mech. Ageing Dev..

[18] Graf C. (2008). The Lawton Instrumental Activities of Daily Living Scale. Am. J. Nurs..

[19] Rolfson D.B., Majumdar S.R., Tsuyuki R.T., Tahir A., Rockwood K. (2006). Validity and Reliability of the Edmonton Frail Scale. Age Ageing.

[20] Tinetti Performance Oriented Mobility Assessment (POMA)*. https://www.leadingagemn.org/assets/docs/Tinetti-Balance-Gait--POMA.pdf.

[21] Caballero F.F., Miret M., Power M., Chatterji S., Tobiasz-Adamczyk B., Koskinen S., Leonardi M., Olaya B., Haro J.M., Ayuso-Mateos J.L. (2013). Validation of an Instrument to Evaluate Quality of Life in the Aging Population: WHOQOL-AGE. Health Qual. Life Outcomes.

[22] Yesavage J.A. (1988). Geriatric depression scale. Psychopharmacol. Bull.

[23] Basińska M.A., Przyborowska-Stankiewicz S., Kruczek A., Liebert A. (2019). Reflective-Ruminative Tendencies and Coping Flexibility in Patients with Non-Specific Inflammatory Bowel Diseases. Adv. Psychiatry Neurol./Postępy Psychiatr. I Neurol..

[24] Liu J., Zhu Y., Tan J.K., Ismail A.H., Ibrahim R., Hassan N.H. (2024). Factors Associated with Frailty in Older Adults in Community and Nursing Home Settings: A Systematic Review with a Meta-Analysis. J. Clin. Med..

[25] Neyens J., Halfens R., Spreeuwenberg M., Meijers J., Luiking Y., Verlaan G., Schols J. (2013). Malnutrition Is Associated with an Increased Risk of Falls and Impaired Activity in Elderly Patients in Dutch Residential Long-Term Care (LTC): A Cross-Sectional Study. Arch. Gerontol. Geriatr..

[26] Puzianowska-Kuznicka M., Januszkiewicz-Caulier J., Kurylowicz A., Mossakowska M., Zdrojewski T., Szybalska A., Skalska A., Chudek J., Franek E. (2021). Prevalence and Socioeconomic Predictors of Diagnosed and Undiagnosed Diabetes in Oldest-Old and Younger Caucasian Seniors: Results from the PolSenior Study. Endokrynol. Pol..

[27] Donini L.M., Poggiogalle E., Molfino A., Rosano A., Lenzi A., Rossi Fanelli F., Muscaritoli M. (2016). Mini-Nutritional As-sessment, Malnutrition Universal Screening Tool, and Nutrition Risk Screening Tool for the Nutritional Evaluation of Older Nursing Home Residents. J. Am. Med. Dir. Assoc..

