# Supplementary material for: Occurrence of Malnutrition among Seniors in Poland Depending on the Place of Residence: An Analysis of Socioeconomic and Health Risk Factors
Source: Nutrients. 2024 Oct 6;16(19):3394. doi: 10.3390/nu16193394 (PMC11478439; doi:10.3390/nu16193394)
Supplement: Supplementary file 1 [file nutrients-16-03394-s001.zip › nutrients-3179688-supplementary.pdf]

Table S1. Nutritional status and place of residence in correlation with functional fitness level - multifactorial variance analysis. **M** represents the mean, or the average value of the variable. **SD** is the standard deviation, indicating the variability or dispersion of the data around the mean. **F** is the F-statistic from the analysis of variance (ANOVA), used to determine whether there are significant differences between group means. **p** is the p-value, which shows the statistical significance of the results (values less than 0.05 are considered significant). **η^2** refers to the effect size (partial eta-squared), which measures the proportion of variance in the dependent variable explained by the independent variable(s). Finally, the **Post-hoc** tests compare specific group pairs to determine which pairs are significantly different, with comparisons indicated as **A < B < C** or **I < II** representing the relative rankings between groups. This applies to all tables S1-S5.

		M	*SD*	*F*	*p*	η^2	*Post-hoc*
A	Malnutrition	17.97	6.12	49.87	0.000	0.240	A < B
B	Risk of malnutrition	21.82	6.93				A < C
C	Normal nutritional status	28.38	5.90				B < C
I	Community-dwelling	27.43	7.22	2.61	0.107	0.006	n.i.
II	Long-term care facility	23.54	6.72				
I.A	Community-dwelling - malnutrition	17.15	6.27	3.74	0.025	0.018	II.C < I.C
I.B	Community-dwelling - risk of malnutrition	23.33	7.13				I.A < I.B
I.C	Community-dwelling - normal nutritional status	29.92	5.34				I.A < I.C
II.A	Facility - malnutrition	19.23	5.89				I.B < I.C
II.B	Facility - risk of malnutrition	20.96	6.74				II.A < II.C
II.C	Facility - normal nutritional status	25.84	5.92				II.B < II.C

Table S2. Nutritional status and place of residence in correlation with the level of frailty - multifactorial variance analysis.

		M	*SD*	*F*	*p*	η^2	*Post-hoc*
A	Malnutrition	9.71	3.15	67.30	0.000	0.301	B < A
B	Risk of malnutrition	7.53	2.79				C < A
C	Normal nutritional status	4.06	2.85				C < B
I	Community-dwelling	4.50	3.65	5.70	0.018	0.013	I < II
II	Long-term care facility	6.63	2.95				
I.A	Community-dwelling - malnutrition	9.74	3.66	2.67	0.071	0.012	I.C < II.C
I.B	Community-dwelling - risk of malnutrition	6.88	3.31				I.B < I.A
I.C	Community-dwelling - normal nutritional status	3.25	2.66				I.C < I.A
II.A	Facility - malnutrition	9.67	2.27				I.C < I.B
II.B	Facility - risk of malnutrition	7.87	2.44				II.C < II.A
II.C	Facility - normal nutritional status	5.39	2.67				II.C < II.B

Table S3. Nutritional status and place of residence in correlation with overall balance and gait - multifactorial variance analysis.

		M	SD	F	p	η^2	Post-hoc
A	Malnutrition	9.30	6.80	39.66	0.000	0.193	A < C
B	Risk of malnutrition	9.53	7.36				B < C
C	Normal nutritional status	18.51	8.14				
I	Community-dwelling	18.87	8.16	20.45	0.000	0.050	II < I
II	Long-term care facility	11.01	7.84				
I.A	Community-dwelling - malnutrition	10.05	7.80	4.45	0.012	0.022	II.B < I. B
I.B	Community-dwelling - risk of malnutrition	12.11	7.72				II.C < I.C
I.C	Community-dwelling - normal nutritional status	21.72	6.28				I.A < I.C
II.A	Facility - malnutrition	8.15	4.95				I.B < I.C
II.B	Facility - risk of malnutrition	7.98	6.75				II.A < II.C
II.C	Facility - normal nutritional status	13.26	8.14				II.B < II.C

Table S4. Nutritional status and place of residence in correlation with depression - multifactorial variance analysis.

		M	SD	F	p	η^2	Post-hoc
A	Malnutrition	7.06	3.70	19.88	0.000	0.114	B < A
B	Risk of malnutrition	5.45	3.50				C < A
C	Normal nutritional status	3.58	2.86				C < B
I	Community-dwelling	4.09	3.54	0.04	0.849	0.000	n.i.
II	Long-term care facility	4.75	3.01				
I.A	Community-dwelling - malnutrition	7.42	3.88	1.31	0.271	0.008	n.i.
I.B	Community-dwelling - risk of malnutrition	5.56	4.17				
I.C	Community-dwelling - normal nutritional status	3.28	2.93				
II.A	Facility - malnutrition	6.54	3.50				
II.B	Facility - risk of malnutrition	5.38	3.10				
II.C	Facility - normal nutritional status	4.06	2.68				

Table S5. Nutritional status and place of residence in correlation with quality of life - multifactorial variance analysis.

		M	*SD*	*F*	*p*	η^2	*Post-hoc*
A	Malnutrition	36.23	6.91	31.38	0.000	0.181	A < C
B	Risk of malnutrition	37.96	6.99				B < C
C	Normal nutritional status	44.62	6.98				
I	Community-dwelling	42.90	7.96	1.90	0.169	0.005	n.i.
II	Long-term care facility	41.39	7.42				
I.A	Community-dwelling - malnutrition	33.62	7.14	2.38	0.095	0.014	I.A < II.A
I.B	Community-dwelling - risk of malnutrition	37.63	7.09				I.A < I.C
I.C	Community-dwelling - normal nutritional status	45.02	6.96				I.B < I.C
II.A	Facility - malnutrition	38.85	5.81				II.A < II.C
II.B	Facility - risk of malnutrition	38.13	7.01				II.B < II.C
II.C	Facility - normal nutritional status	43.97	7.01				
